# Evaluation of risk factors for a fulminant Clostridium difficile infection after cardiac surgery: a single-center, retrospective cohort study

**DOI:** 10.1186/s12871-018-0597-2

**Published:** 2018-09-27

**Authors:** Maximilian Vondran, Senta Schack, Jens Garbade, Christian Binner, Meinhard Mende, Ardawan Julian Rastan, Michael Andrew Borger, Thomas Schroeter

**Affiliations:** 1 University Department for Cardiac Surgery, Heart Center Leipzig, Struempellstr. 39, 04289 Leipzig, Germany; 2 Department of Cardiovascular Surgery, Thoracic and Vascular Surgery, University Hospital Marburg (UKGM), Baldingerstr, 35043 Marburg, Germany; 3 Department of Cardiac Surgery, Center of Cardiovascular Diseases Rotenburg a. d. Fulda, Heinz-Meise-Str. 100, 36199 Rotenburg a. d. F, Germany; 4 Center for Clinical Trials, University of Leipzig, Härtelstraße 16/18, 04107 Leipzig, Germany

**Keywords:** Intensive care, Clostridium difficile, Cardiac surgery, High risk, Critical care

## Abstract

**Background:**

Clostridium difficile (CD) is the most common pathogen causing nosocomial diarrhea. The clinical presentation ranges from mild diarrhea to severe complications, including pseudomembranous colitis, toxic megacolon, sepsis, and multi-organ failure. When the disease takes a fulminant course, death ensues rapidly in severe and complex cases. Preventive screening or current prophylactic therapies are not useful. Therefore, this study was conducted to detect risk factors for a fulminant CD infection (CDI) in patients undergoing cardiac surgery.

**Methods:**

Between April 1999 and April 2011, a total of 41,466 patients underwent cardiac surgery at our institution. A review of our hospital database revealed 1256 patients (3.0%) with post-operative diarrheal disease who tested positive for CD; these patients comprised the cohort of this observational study. A fulminant CDI occurred in 153 of these patients (12.2%), which was diagnosed on the basis of gastrointestinal complications, e.g. pseudomembranous colitis, and/or the need for post-cardiac surgery laparotomy. Demographic, peri-operative, and survival data were analyzed, and predictors of a fulminant CDI were assessed by binary logistic regression analysis.

**Results:**

The 30-day mortality was 6.1% (*n* = 77) for the entire cohort, with significantly higher mortality among patients with a fulminant CDI (21.6% vs. 4.0%, *p* <  0.001). Overall mortality (27.7%, *n* = 348) was also higher for patients with a fulminant course of the disease (63.4% vs. 22.8%, *p* <  0.001), and a laparotomy was required in 36.6% (*n* = 56) of the fulminant cases. Independent predictors of a fulminant CDI were: diabetes mellitus type 2 (OR 1.74, CI 1.15–2.63, *p* = 0.008), pre-operative ventilation (OR 3.52, CI 1.32–9.35, *p* = 0.012), utilization of more than 8 units of red blood cell concentrates (OR 1.95, CI 1.01–3.76, *p* = 0.046) or of more than 5 fresh-frozen plasma units (OR 3.38, CI 2.06–5.54, *p* <  0.001), and a cross-clamp time > 130 min (OR 1.93, CI 1.12–3.33, *p* = 0.017).

**Conclusions:**

We identified several independent risk factors for the development of a fulminant CDI after cardiac surgery. Close monitoring of high-risk patients is important in order to establish an early onset of therapy and thus to prevent a CDI from developing a fulminant course after cardiac surgery.

## Background

In recent years, the bacterium Clostridium difficile (CD), a gram-positive, spore-forming, anaerobic bacilli that has been known for almost a century, has played an increasingly important role in postoperative infections. There is currently a growing incidence of diarrhea caused by CD, and especially after the administration of antibiotics about 10% to 20% of all diarrheal cases can be attributed to CD [1]. Subclinical colonization with CD is not at all uncommon; for example, it is estimated that up to 80% of children may be affected [2]. This pathogen is the most common cause of nosocomial diarrhea [3, 4]. The incidence of CD-associated diarrhea after various surgical procedures varies from 0.3 to 8.4% [5] in the current literature, depending on the surgical cohort investigated, and mild to moderate severity of CD-associated diarrhea translates into a longer hospital stay. A fulminant course, however, with pseudomembranous colitis and severe complications such as toxic megacolon, including intestinal perforation with sepsis and multi-organ failure, significantly increases mortality after surgical procedures [6]. Despite knowledge gained about CD as one of the most important nosocomial pathogens and extensive investigation of its influence on patients in nursing homes, little is known about the incidence and risk factors for the development of a CD infection (CDI) after surgical interventions, especially in cardiac surgery.

Closer inspection reveals that cardiac surgical patients in particular suffer from many of the prerequisites for colonization with CD with regard to the known predisposition factors, including antibiotic prophylaxis, age, diabetes mellitus, renal or cardiac insufficiency, use of blood products, duration of extracorporeal circulation and ischemic period, and length of stay on an intensive care unit (ICU) [5, 7–10].

There are a few reports available that address CDI and clinical outcomes following cardiac surgery [5, 8–10]. However, there is no study to date that focuses on CDI with a fulminant course. The purpose of this single-center, retrospective study was to identify risk factors for the development of a fulminant CDI after cardiac surgery. Additional attention was given to the clarification of the incidence and mortality of bland and fulminant CDI and the clinical outcomes of the patients during follow-up.

## Methods

### Patients

Between April 1999 and April 2011, 41,466 patients underwent cardiac surgery at our center. Demographics and pre-, intra-, and post-operative data were collected prospectively for all patients in a digital hospital registry. A review of this registry revealed that 1256 out of 41,466 (3.0%) patients were diagnosed with a CDI during their hospital stay. The presence of a CDI was verified using medical records, discharge summaries from other institutions, and documentation of findings from other departments. The local ethics committee approved the study (University of Leipzig, Az.: 212–15-01062015). The study design, anonymous data acquisition, and the publication of the data were in accordance with the Declaration of Helsinki.

### Diagnosis of CDI

CDI was diagnosed by a combination of clinical symptoms and laboratory testing. The criteria for a CDI were frequent stool (> 3 times/day) with unformed consistency (water content of the stool > 75%), increased stool masses (> 250 g/day), and stool testing positive for CD [11]. The stool examination was made with cytotoxicity assay and/or enzyme immunoassay for CD toxins. All patients who fulfilled these criteria were included in this retrospective cohort study (*n* = 1256). Patients were excluded if they met any of the following criteria: age < 18 years, a hospital stay of less than 48 h, and positive test for CD at the time of hospital admission. The occurrence of gastrointestinal complications (e.g. pseudomembranous colitis, toxic megacolon, gastrointestinal bleeding, ileus, ischemia, or perforation, etc.) and/or the need for a laparotomy after cardiac surgery were defined as criteria for a fulminant CDI (Table 1).

**Table 1:** Gastrointestinal complications

Variable	CDI positive *n* = 1256	
	n	%
Laparotomy	56	4.5
Gastrointestinal bleeding	54	4.3
Acute abdomen and/or peritonitis and/or perforation	20	1.6
Ileus/ischemia	23	1.8
Pseudomembranous colitis	35	2.8
Toxic megacolon	7	0.6
Other abdominal complications	20	1.6

Abbreviations: *CDI* Clostridium difficile infection

### Statistics

Unless otherwise indicated, categorical variables are presented as numbers and percentage, and continuous parameters are expressed as mean ± standard deviation (SD). Fisher’s exact test or a Chi-squared test was used to assess differences for categorical variables, and Student’s t-test or a Wilcoxon rank-sum test was applied for continuous parameters. Overall survival rates were analyzed using the Kaplan-Meier method. Statistical differences were calculated by the log-rank test. Multivariate analysis was performed using a binary logistic regression model (stepwise backward) to differentiate independent risk factors for developing a fulminant course of CDI. Variables used in the equation were prior cardiac surgery, pre-operative dialysis, pre-operative ventilation, pre-operative low cardiac output, emergency surgery, minimally invasive surgery, diabetes mellitus type 2, age > 80 years, left ventricular ejection fraction (LVEF) < 30%, New York Heart Association (NYHA) class ≥III, length of surgery > 200 min, cross-clamp time > 130 min, > 8 units of red blood cell (RBC) concentrates, > 5 units of fresh-frozen plasma (FFP).

All tests were two-sided, with a significance level alpha = 0.05. Unless otherwise stated the analyses were performed by IBM SPSS Statistics for Macintosh, Version 24.0 (IBM Corp., Armonk, NY, USA). All authors had full access to datasets and upheld their integrity. They read and agreed to the manuscript as written.

## Results

Table 2 summarizes all pre-operative patient characteristics. A total of 1256 patients (3.0% of cardiac surgery patients) developed a post-operative CDI between April 1999 and April 2011. Of these, 153 (12.2%) developed a fulminant CDI during their stay. The patients with a fulminant CDI had a significantly lower pre-operative LVEF than that of the group with a bland CDI. Furthermore, the pre-operative proportion of patients with diabetes mellitus, LVEF < 30%, age > 80 years, prior cardiac surgery, ventilator dependence, and a poor NYHA functional class was significantly higher for the group developing a fulminant CDI.

**Table 2:** Patient characteristics

Variable	All CDI patients *n* = 1256		Bland CDI *n* = 1103		Fulminant CDI *n* = 153		*p*-Value
	Mean	SD	Mean	SD	Mean	SD	
Age, y	72.2	10.5	72.3	10.6	71.6	10.1	0.444
Left ventricular ejection fraction, %,	53.9	14.5	54.4	14.3	51.0	15.8	0.013
Creatinine, mg/dl	1.22	1.03	1.21	1.02	1.27	1.08	0.484
	n	%	n	%	n	%	
Male	732	58.3	631	57.2	101	66.0	0.440
Age > 80 years	246	19.6	227	20.6	19	12.8	0.027
LVEF < 30%	112	8.9	90	8.2	22	14.4	0.015
Diabetes mellitus	468	37.3	396	35.9	72	47.1	0.009
Coronary heart disease	675	53.7	596	54.0	79	51.6	0.858
Prior acute myocardial infarction	313	24.9	272	24.7	41	26.8	0.551
Peripheral artery disease	355	28.3	319	28.9	36	23.5	0.180
Pre-operative dialysis	66	5.3	53	4.8	13	8.5	0.078
Hypertension	1053	83.8	930	84.3	123	80.4	0.241
PAP ≥60 mmHG	101	8.0	87	7.9	14	9.2	0.633
Chronic obstructive pulmonary disease	127	10.1	108	9.8	19	12.4	0.317
Hyperlipidemia	657	52.2	581	52.7	75	49	0.437
Nicotine abuse	352	28.0	317	28.7	35	22.9	0.150
Prior cardiac surgery	161	12.8	128	11.6	33	21.6	0.001
Prior malignant disease	78	6.2	69	6.3	9	5.9	1.000
Pre-operative neurological dysfunction	107	8.5	92	8.3	15	9.8	0.537
Pre-operative ventilation	21	1.7	12	1.1	9	5.9	< 0.001
Pre-operative any mechanical circulatory assist device	32	2.5	26	2.4	6	4.0	0.477
NYHA class							
I	93	7.4	88	8.0	5	3.3	
II	330	26.3	297	26.9	33	21.6	
III	644	51.3	559	50.7	85	55.6	
IV	189	15.0	159	14.4	30	19.6	0.036
≥ III	189	15.0	159	14.4	30	19.6	0.116
Body mass index ≥25.0 g/m^2^	830	66.1	725	65.8	105	68.6	0.478

Abbreviations: *CDI* Clostridium difficile infection

Table 3 summarizes all peri-procedural details. The duration of surgery, cardiopulmonary bypass (CPB) time, cross-clamp time, and time of reperfusion were significantly longer for the patients with a fulminant CDI. The percentage of patients needing emergency surgery was also significantly higher in the group with a fulminant CDI, whereas minimally invasive surgery was performed significantly more often in the group with bland CDI.

**Table 3:** Procedural details

Variable	All CDI patients *n* = 1256		Bland CDI *n* = 1103		Fulminant CDI *n* = 153		*p*-Value
	Mean	SD	Mean	SD	Mean	SD	
Length of surgery, min	194.8	91.6	191.3	89.4	220.7	103.3	< 0.001
CPB time, min	127.6	55.7	124.8	53.1	146.0	67.8	0.001
Cross-clamp time, min	82.9	34.7	81.3	33.5	94.4	40.5	< 0.001
Time of reperfusion, min	31.7	24.3	30.8	22.9	38.3	32.0	0.020
Time of circulatory arrest, min	34.3	22.5	33.8	23.9	36.1	17.3	0.808
	n	%	n	%	n	%	
Emergency surgery	144	11.5	113	10.2	31	20.3	0.001
Length of surgery > 200 min	546	43.5	465	42.2	81	52.9	0.015
Cross-clamp time > 130 min	503	40.1	420	38.0	83	54.0	0.001
Surgery performed							
Valve	550	43.8	485	44.0	65	42.5	0.728
CABG	244	19.4	212	19.2	32	20.9	0.619
Aortic	31	2.5	26	2.4	5	3.3	0.496
Others or combination	431	34.4	380	34.5	51	33.3	0.785
Any ablation for AF	170	13.5	152	13.8	18	11.8	0.614
Intra-operative any mechanical circulatory assist device	67	5.3	52	4.7	15	9.8	0.070
Minimally invasive surgery	424	33.8	389	35.3	35	22.9	0.009
CPB	950	75.6	826	74.9	124	81.0	0.071
Circulatory arrest	34	2.7	27	2.4	4	4.6	0.129

Abbreviations: *AF* Atrial fibrillation, *CABG* Coronary artery bypass grafting, *CDI* Clostridium difficile infection, *CPB* Cardiopulmonary bypass

Table 4 summarizes post-operative outcomes. The post-operative course of the patients with a fulminant CDI was associated with more complications. This group required mechanical circulatory assist devices (MCAD) significantly more often. Whereas the bland CDI group needed MCAD in just 4.6% (*n* = 51) of cases, MCAD was required in 19.0% (*n* = 51) of patients with a fulminant course. The proportion of intra-aortic balloon pump (IABP) support as MCAD was 93.1% in the fulminant CDI group and 80.4% in the bland CDI group. (*p* = 0.221). Moreover, renal replacement therapy, re-intubation, and a tracheostomy was required significantly more often in the group with fulminant CDI. Furthermore, post-operative neurological dysfunction was diagnosed more frequently in the patients with a fulminant CDI. The utilization of RBC concentrates, FFP units, and platelet concentrates was significantly greater for the patients with a fulminant CDI, and these patients were ventilated longer and the length of ICU or intermediate care unit stay was significantly longer.

**Table 4:** Postoperative Outcomes

Variable	CD positive *n* = 1256		Bland CDI *n* = 1103		Fulminant CDI *n* = 153		*p*-Value
	Mean	SD	Mean	SD	Mean	SD	
RBC, units	8.0	14.1	6.44	11.9	19.2	21.6	< 0.001
FFP, units	5.3	13.2	3.8	10.0	16.3	24.2	< 0.001
Platelets, units	1.2	3.8	0.9	3.4	3.4	5.6	< 0.001
Ventilation time up to first extubation, h	81.6	225.3	66.3	187.1	187.0	383.8	< 0.001
Total ventilation time, h	181.4	359.7	1384	289.8	477.6	585.6	< 0.001
Total LOS ICU, h	282.3	430.9	234.7	364.5	586.9	647.4	< 0.001
Total LOS IMCU, h	202.8	380.5	188.5	372.5	319.6	424.6	0.002
	n	%	n	%	n	%	
Post-operative any mechanical circulatory assist device	80	6.4	51	4.6	29	19.0	< 0.001
Post-operative dialysis	301	24.0	221	20.0	80	52.3	< 0.001
Re-intubation	370	29.5	286	25.9	84	54.9	< 0.001
Tracheostomy	197	15.7	135	12.2	62	40.5	< 0.001
Re-thoracotomy	169	13.5	146	13.2	23	15.0	< 0.001
Post-operative neurological dysfunction	948	75.5	817	74.1	131	85.6	0.002
> 8 units RBC	750	59.7	616	55.8	134	87.6	< 0.001
> 5 units FFP	477	38.0	366	33.2	111	72.5	< 0.001
> 2 units platelets	144	11.5	98	8.9	46	30.1	< 0.001

Abbreviations: *FFP* Fresh-frozen plasma, *ICU* Intensive care unit, IMCU Intermediate care unit, *LOS* Length of surgery, *RBC* Red blood cells

**Fig. 1:**
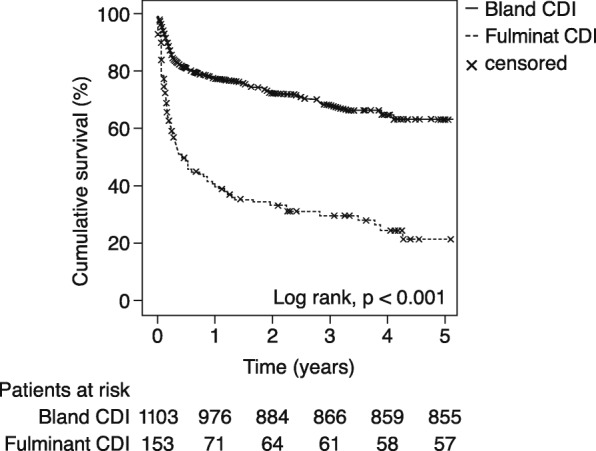
Cumulative survival of patients with bland CDI vs. fulminant CDI

The 30-day all-cause mortality was 6.1% (*n* = 77) and overall all-cause mortality was 27.7% (*n* = 348) for the entire study cohort. Patients with a fulminant CDI had a significantly higher 30-day all-cause mortality rate than patients with a bland CDI (21.6% vs. 4.0%, *p* <  0.001). Moreover, the overall all-cause mortality was significantly higher for patients with a fulminant CDI (63.4% vs. 22.8%, *p* <  0.001; Fig. 1). The median follow-up time was 8 (interquartile range: 2; 30) months. Diabetes mellitus type 2, pre-operative ventilation, utilization of more than 8 RBC concentrates, more than 5 FFP units, and a cross-clamp time > 130 min were multiple independent predictors of a fulminant CDI (Table 5).

**Table 5:** Results of the logistic regression analysis

Variable	Odds Ratio	95% CI		*p*-Value
Diabetes mellitus	1.743	1.154	2.633	0.008
Pre-operative ventilation	3.517	1.323	9.353	0.012
Cross-clamp time > 130 min	1.934	1.123	3.329	0.017
> 8 units RBC	1.952	1.012	3.764	0.046
> 5 units FFP	3.382	2.063	5.544	< 0.001

Abbreviations: *CI* Confidence interval, *FFP* Fresh-frozen plasma, *RBC* Red blood cells

## Discussion

Post-operative diarrhea is a common, underestimated complication after surgery that can be caused by CD. While the majority of CDI cases are harmless, in 8–16% a fulminant CDI with severe complications occurs [12–14] that significantly increases mortality after surgical procedures [6]. Little data exists on the incidence and risk factors for the development of a CDI after cardiac surgery, especially for fulminant CDI. The present study is the first to address fulminant CDI after cardiac surgery. The findings of this study can be summarized as follows: 12.2% of the CDI cases in our cohort were fulminant; fulminant CDI increases 30-day and overall all-cause mortality significantly compared with bland CDI; independent risk factors for a fulminant CDI are pre-operative diabetes mellitus type 2, pre-operative ventilation, cross-clamp time > 130 min, the utilization of more than 8 RBC units or more than 5 FFP units.

### Incidence

The incidence of post-operative CDI varies in the current literature and often depends on the surgery performed, decade of the study, and the study design. It ranges from 0.3 to 8.4% [5]. The most recent data from studies of cardiac surgery dealing with the topic of post-surgical CDI show very low incidences, ranging between 0.3–0.79%, with a tremendous increase worldwide a few years after the beginning of the new millennium [5, 8, 9]. Our cohort seems to reflect more the data of a real-world cohort, with an overall incidence of 3.0%. However, Crabtree et al. reported that in June 2003 their highest incidence was 8.89% [5]. Since 2003, not only an increase in the incidence of CDI but also the severity of the disease has been reported worldwide [15]. In Germany, an analysis of the discharge diagnoses of the years 2000–2004 showed a clear increase in CDI from 7 to 39 cases per 100,000 inpatients; between 2004 and 2006 it doubled again [16, 17]. In the context of clusters that first appeared in North America and then in Europe, including Germany, a new epidemic strain with particular virulence has been reported [15, 18].

### Mortality

Current data on 30-day mortality in patients with CDI vary between 2.5 and 26% [8, 9, 19, 20], depending on the study population. Thus, a 30-day mortality rate of 26% reported by Musa et al. was based on a collective of only 27 ICU patients after cardiothoracic surgery [20]. Keshavamurthy and colleagues published an in-hospital mortality rate of 11% in 2014 for a cohort of 145 patients who underwent cardiac surgery at the Cleveland Clinic [9]. Flagg et al. analyzed 349,122 patients after cardiac surgery from 2004 to 2008 from the Nationwide Inpatient Sample database. The in-hospital mortality of their cohort of CDI patients was 12% [8]. Both studies reported a significantly higher 30-day mortality for patients with CDI after cardiac surgery, whereas Crabtree et al. found similar survival rates between different surgical groups [5]. Lemaire et al. showed in their study that with a post-operative CDI, the risk of in-hospital mortality nearly doubles [10]. In the present study, a 30-day mortality of 4.0% was observed for the group with bland CDI and 21.6% for the group with fulminant disease. At this point, it should be emphasized how clearly the 30-day mortality of the patients with fulminant disease is increased compared with those with bland CDI. With more than 20% mortality in the first 30 days, a fulminant CDI must be viewed as a highly life-threatening condition.

Reports of overall or long-term mortality following a CDI have yielded various results. A retrospective study by Morrison et al. reported an overall mortality rate of 5%, whereas Keshavamurthy found a 3-year mortality in their CDI cohort as high as 48% [9, 21]. Crabtree et al. described an overall mortality of 6% for a cohort of post-operative CDI patients, although the overall mortality rate for the subgroup with fulminant CDI was 30% [5]. Further studies reported mortality rates for the group of patients with fulminant CDI of up to 50% and up to 80% in cases requiring emergency colectomy [21, 22]. In the present study we observed an overall mortality in the collective with bland CDI of 22.8%. In contrast, in the group with a fulminant course, the overall mortality was 63.4%. Moreover, most of the deaths occurred in the first 3 months after cardiac surgery, whether the CDI was fulminant or bland.

### Risk factors for the development of a fulminant CDI

Cardiopulmonary comorbidities have been repeatedly documented in the literature as risk factors for the development of fulminant CDI [23, 24]. The present work underpins these observations. Reduced LVEF and elevated NYHA functional class are dependent risk factors and pre-operative mechanical ventilation is an independent risk factor for the development of a fulminant CDI. Likewise, prior cardiac surgery in the present study was a dependent risk factor for the development of a fulminant CDI. Thus, both idiopathic and iatrogenic cardiopulmonary factors create a vulnerable niche for severe CDI [25].

Also worthy of mention is the relationship between severe disease progression and the indication for surgery. Patients admitted for emergency procedures showed significantly more fulminant events than patients undergoing elective surgery. This concurs with the previously published studies and suggests that the toxicity of the pathogen in already pre-operatively weakened patients with a higher rate of circulatory instability results in far graver outcomes than in elective patients with a lower peri-operative stress response [10].

As mentioned above, pre-operative mechanical ventilation is a further independent risk factor for the development of a fulminant CDI. In general, a patient who has been ventilated before cardiac surgery is rarely in good clinical condition. Here, the body is exposed to a high degree of stress even before the surgical procedure. Furthermore, ventilation itself can contribute additional immunosuppression [26], which may support a fulminant CDI.

The peri-operative administration of blood products has been frequently associated in the literature with an aggravation of the course of the disease and with modulation of recipients’ immune function [5, 20, 27]. The present work underlines these observations: peri-operative administration of more than eight RBC units or five FFP units increased the risk of a fulminant course by two to three times. A controversial issue here is whether the administration of blood products is a true, aggravating factor or rather a harbinger of the increasing severity of the disease.

The available data on the influence of peri-operative MCAD on the incidence and severity of CDI is very scarce [20], but the use of these devices seems to have an effect on outcome [28]. The present study shows an influence of the post-operative use of a MCAD on the course of a CDI. In the vast majority of our cases, this was the implantation of an IABP in hemodynamically unstable patients. The risk of developing a fulminant CDI was increased in patients who were treated with a MCAD. Importantly, a previous study has shown that up to 97% of implanted IABP devices may compromise the blood flow of the visceral arteries by overlapping them [29]. About 25% of cases become clinically symptomatic, show intestinal ischemia, and/or have to be treated by laparotomy. In addition to the existing infection and hemodynamic instability, the gastrointestinal tract is especially vulnerable due to compression of visceral arteries by the IABP and consequently reduced perfusion [30]. This is a possible explanation for the significantly increased rate of gastrointestinal complications and visceral surgical interventions in the collective of patients treated with a MCAD.

The duration of surgery and CPB were found to be dependent risk factors, and the cross-clamp time was an independent risk factor for the development of fulminant CDI. Crabtree et al. were also able to demonstrate the influence of prolonged surgical duration with prolonged CPB time on the development of CDI and protraction of underlying disease [5]. The present study also shows that in patients who underwent surgery through a minimally invasive approach, the risk of developing fulminant versus bland CDI was reduced. Thus, minimally invasive surgery was a univariate protective predictor against a fulminant CDI. The clear advantage of minimally invasive surgery is the less traumatic procedure due to smaller wounds and less peri-operative blood loss as well as shorter length of stay on the ICU or intermediate care unit. The present study demonstrated that the length of stay in intensive and intermediate care were dependent risk factors for developing a fulminant CDI, which was also described in previous investigations [5, 20, 31]. These results should be interpreted with caution, however, as causality of the relationships may be open to discussion. Therefore, the statistical categorization between cause and effect of the phenomenon regarding the CDI and the ICU/intermediate care unit stay is difficult. In summary, a prolonged hospital stay is most likely both a risk factor for the development of a fulminant CDI and a consequence of it.

### Limitations

In the present work, no ribotyping of the CD strains was performed; therefore, possible influences of hypervirulent strains could not be independently evaluated. Due to the retrospective study design, it was no longer possible to determine leukocyte counts, lactate values, and creatinine levels of the patients at the time of infection. However, these parameters were frequently used in the literature to classify the severity of CDI [32]. In this work, only the development of gastrointestinal complications and the need for laparotomy could be used; thus, such a classification was not possible. The content of an RBC, FFP and platelet concentrate unit varies and was not individually recorded in our work, as only the administered units have been documented. The volume of one unit was appx. 250 ml. Furthermore, we did not have sufficient information about the other hospitals or nursing homes from which the patients were transferred who were admitted to the Heart Center Leipzig. The retrospective study design also prohibited us from clearly reconstructing in all patients whether antibiotics had already been administered pre-operatively or not. It is well known that antibiotics cause CDI, but they are also components of optimal therapy of fulminant CDI. Because of this and the unstable data, we did not include antibiotic therapy as such in our risk stratification model. Finally, the relapse rate was not determined in the present study. There are indications in the literature that more than one third of CDI patients experience at least one relapse [14].

## Conclusions

In the present study we were able to detect independent risk factors for the development of a fulminant CDI. It is striking that the multivariate predictors would all appear to have a negative effect on the immune system or in some way to lower immune system defenses via stress on the patient’s body. The severity of the course of CDI, in our view, depends on the vulnerability of the immune system and the stimuli that compromise it. A disturbance of the intestinal physiology and thus the microbial intestinal flora, e.g. by gastrointestinal diseases, surgical interventions, or antibiotic treatment, plays the biggest role here. Since preventive screening or prophylactic therapies are not useful, high-risk patients can be selected and targeted for close monitoring and an early intervention to avoid a fulminant course of an infection. By providing knowledge about peri-operative risk factors, this work can be a step towards the establishment of consistent risk stratification systems. Until new and effective treatment concepts are established, the selection of particularly vulnerable patients and their targeted observation is a way to control fulminant CDI of nosocomial origin.

## Abbreviations

AF: Atrial fibrillation
CABG: Coronary artery bypass grafting
CD: Clostridium difficile
CDI: Clostridium difficile infection
CPB: Cardiopulmonary bypass
FFP: Fresh-frozen plasma
IABP: Intra-aortic balloon pump
ICU: Intensive care unit
IMCU: Intermediate care unit
LOS: Length of surgery
LVEF: Left ventricular ejection fraction
MCAD: Mechanical circulatory assist devices
NYHA: New York Heart Association
RBC: Red blood cells
SD: Standard deviation
